# Transcriptome alterations in myotonic dystrophy frontal cortex

**DOI:** 10.1016/j.celrep.2020.108634

**Published:** 2021-01-19

**Authors:** Brittney A. Otero, Kiril Poukalov, Ryan P. Hildebrandt, Charles A. Thornton, Kenji Jinnai, Harutoshi Fujimura, Takashi Kimura, Katharine A. Hagerman, Jacinda B. Sampson, John W. Day, Eric T. Wang

**Affiliations:** 1 Department of Molecular Genetics & Microbiology, Center for NeuroGenetics, Genetics Institute, University of Florida, Gainesville, FL, USA; 2 Department of Neurology, University of Rochester Medical Center, Rochester, NY, USA; 3 Department of Neurology, National Hospital Organization Hyogo-Chuo Hospital, Sanda, Japan; 4 Department of Neurology, National Hospital Organization Toneyama Hospital, Osaka, Japan; 5 Department of Neurology, Hyogo College of Medicine, Nichinomiya, Japan; 6 Department of Neurology, Stanford University, Palo Alto, CA, USA; 7 These authors contributed equally; 8 Lead contact

## Abstract

Myotonic dystrophy (DM) is caused by expanded CTG/CCTG repeats, causing symptoms in skeletal muscle, heart, and central nervous system (CNS). CNS issues are debilitating and include hypersomnolence, executive dysfunction, white matter atrophy, and neurofibrillary tangles. Here, we generate RNA-seq transcriptomes from DM and unaffected frontal cortex and identify 130 high-confidence splicing changes, most occurring only in cortex, not skeletal muscle or heart. Mis-spliced exons occur in neurotransmitter receptors, ion channels, and synaptic scaffolds, and GRIP1 mis-splicing modulates kinesin association. Optical mapping of expanded CTG repeats reveals extreme mosaicism, with some alleles showing >1,000 CTGs. Mis-splicing severity correlates with CTG repeat length across individuals. Upregulated genes tend to be microglial and endothelial, suggesting neuroinflammation, and downregulated genes tend to be neuronal. Many gene expression changes strongly correlate with mis-splicing, suggesting candidate biomarkers of disease. These findings provide a framework for mechanistic and therapeutic studies of the DM CNS.

## In Brief

Otero et al. characterize DM1 frontal cortex transcriptomes and observe extreme CTG repeat lengths even in individuals showing modest mis-splicing. Mis-splicing is enriched in ion channels, neurotransmitter receptors, and synaptic scaffolds, in some cases potentially altering synaptic trafficking. Inferred expression signatures from microglia and endothelial cells suggest neuroinflammation.

## Graphical Abstract

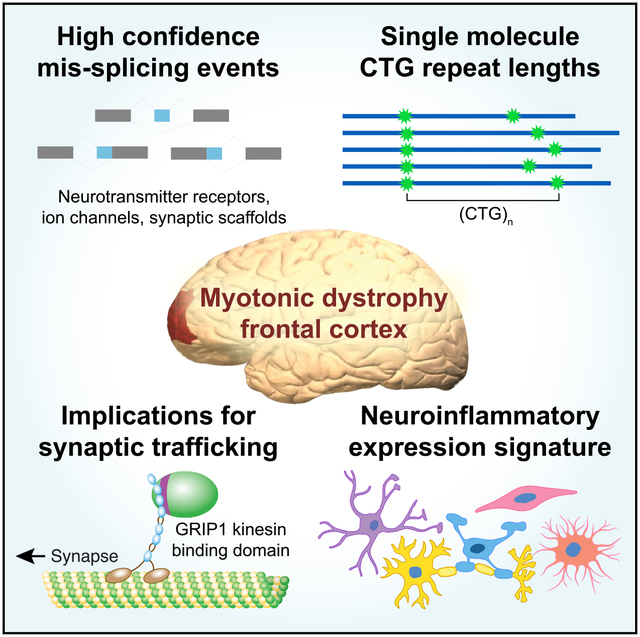

## INTRODUCTION

Myotonic dystrophy (DM) is a multi-systemic, progressive disease caused by expanded CTG or CCTG repeats in the 3′ UTR of the dystrophia myotonic protein kinase (DMPK) gene (DM type 1 [DM1]) (Brook et al., 1992) or the first intron of the cellular nucleic acid binding protein (CNBP) gene (DM type 2 [DM2]) (Liquori et al., 2001), respectively. Both DM1 and DM2 are highly variable in age of onset, clinical features, and disease severity. Although DM is well studied in the context of peripheral symptoms such as myotonia and muscle weakness, central nervous system (CNS) symptoms are also common in DM and can contribute significantly to neurological impairment (Heatwole et al., 2012, 2015). These symptoms include hypersomnia, executive functioning deficits, memory deficits, and emotional disturbances (Modoni et al., 2008; Weber et al., 2010; Schneider-Gold et al., 2015). Imaging studies show white and gray matter abnormalities in multiple brain regions, as well as ventricle enlargement (reviewed in Minnerop et al., 2018). However, the molecular mechanisms driving these neurobiological changes remain largely unknown.

There is strong evidence to support a pathomechanism in which the Muscleblind-like (MBNL) family of RNA binding proteins (RBPs) are functionally depleted in both peripheral nervous system and CNS tissues in DM, resulting in global splicing dysregulation and potentially additional alterations in RNA metabolism (Jiang et al., 2004; Miller et al., 2000; Charizanis et al., 2012; Lee et al., 2013; Goodwin et al., 2015; Nishi et al., 2020). However, limited analyses have been performed to comprehensively identify transcriptome changes in the CNS across a broad subset of patients, assess the extent to which MBNLs are depleted, and address whether other RBPs are perturbed. RNA sequencing (RNA-seq) has not been applied to CNS tissues in DM2, and the extent to which RBFOX proteins may modulate CNS pathogenesis in DM2 (Sellier et al., 2018) is unexplored. Furthermore, as opposed to skeletal muscle, in which dysfunction is predominantly driven by myonuclei, the extent to which different cell types are affected in the CNS of DM-affected individuals is unknown. CUG foci have been observed in neurons, glia, and oligodendrocytes (Jiang et al., 2004), but the contribution of each cell type to pathology has not been extensively explored. Although transcriptome dysregulation has been extensively studied in peripheral DM1 tissues (Wang et al., 2019; Wagner et al., 2016; Du et al., 2010; Freyermuth et al., 2016), the repertoire of mRNAs expressed in the CNS is distinct. Therefore, a different set of exons may be mis-regulated in this tissue. Finally, although there are clear examples of how specific splicing events in peripheral tissues cause particular DM symptoms—Clcn1 and myotonia (Wheeler et al., 2007), Bin1 and muscle weakness (Fugier et al., 2011), and Scn5a and cardiac arrhythmias (Freyermuth et al., 2016)—no clear examples exist in the CNS. To lay the groundwork required to answer some of these questions, here we generate and analyze transcriptomes derived from a set of post-mortem frontal cortex (FC) samples (Brodmann area 10) from DM1 patients, DM2 patients, and unaffected controls. We identify high-confidence mis-spliced exons that show a gradient of changes across patients and study how a mis-splicing event in GRIP1 may alter its efficacy as a synaptic adaptor. We analyze DM transcriptomes, together with additional single-cell transcriptome datasets (Darmanis et al., 2015), to assess potential changes in cell-type composition and determine which cell types are potentially responsible for changes in gene expression and splicing patterns.

Somatic instability is well established as a major driver of age of onset in DM1 and other repeat expansion diseases (Thornton et al., 1994; Schmidt and Pearson, 2016; Morales et al., 2012), but technical challenges have precluded facile, direct assessment of full-length repeat-containing alleles in the DM1 CNS. Furthermore, the CTG repeat lengths required to sequester sufficient MBNL to elicit robust mis-splicing have not been assessed in this tissue. New technologies such as long read sequencing provide some advantages over Southern blotting and small-pool PCR (O’Rourke et al., 2015) but often still require amplification or sub-cloning, which can introduce some biases. Here, we apply an optical mapping approach to size expanded CTG repeats at single-molecule resolution in DM1; this approach allows unbiased, amplification-free measurement of repeat lengths from genomic DNA (Lam et al., 2012; Barseghyan et al., 2017).

Finally, although studies of transcriptomes can identify molecular events driving disease features, they may also suggest potential markers of disease status. Here, we study changes in FC, but similar molecular changes may occur in other brain regions. The aggregate of these pathological processes may be reflected in the composition of cerebrospinal fluid (CSF), facilitating the development of accessible biomarkers to more precisely characterize disease severity and measure changes following therapeutic intervention. Indeed, CSF biomarkers have been developed for other neurological diseases such as Huntington’s disease (HD), C9orf72 amyotrophic lateral sclerosis/frontotemporal dementia (C9ALS/FTD), and Alzheimer’s disease (Silajdžić and Björkqvist, 2018; Gendron et al., 2017; Lehmer et al., 2017; Holtzman, 2011). In parallel, investigators have used proteomics to broadly characterize proteins present in CSF. In DM1, splicing biomarkers correlate with muscle strength (Nakamori et al., 2013) and potentially reflect the concentration of free MBNL in skeletal muscle (Wagner et al., 2016). Subsequent approaches have been developed to profile RNAs present in urine (Antoury et al., 2018), potentially providing a non-invasive route to measure disease status. By integrating our transcriptome analyses with existing knowledge of proteins present in CSF, we lay critical groundwork to properly inform and interpret future biomarker discovery efforts.

## RESULTS

### Splicing dysregulation in DM1 FC exhibits a gradient of severity

**Figure 1.**
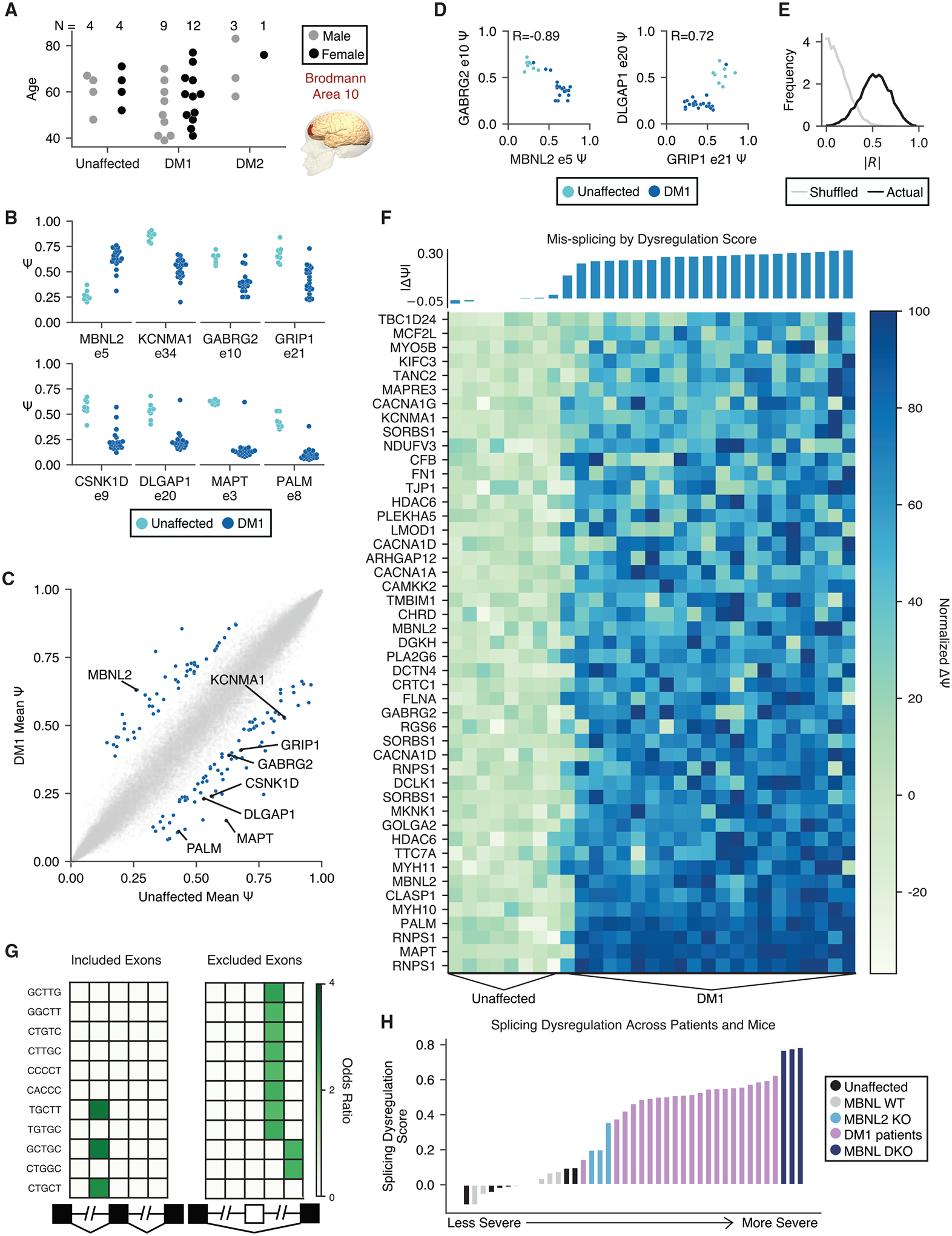
Mis-splicing changes across DM1 FC samples show a gradient of severity consistent with quantitative loss of MBNL (A) RNA-seq was performed on 21 DM1, 4 DM2, and 8 unaffected FC (Brodmann area 10) samples across a range of ages. (B) Percentage spliced in (PSI, ψ) for specific exons in unaffected and DM1 FC samples. (C) Scatterplot of mean ψ for unaffected versus DM1 samples for all exons measured; 130 mis-splicing events were detected as significantly regulated (|Δψ| > 0.2, p < 0.01 by rank-sum test) and are highlighted in blue. (D) Scatterplot of ψ for MBNL2 exon 5 versus GABRG2 exon 10 and GRIP1 exon 21 versus DLGAP1 exon 20 across unaffected and DM1 individuals. The Pearson correlation value is shown. (E) Histogram of correlation values for all pairs of 130 significantly regulated exons (black). A similar histogram of correlation values for all pairs following shuffling of patient identities is also shown (gray) (see STAR Methods). A Kolmogorov-Smirnov (KS) test shows that the distributions are different (p < 1e–300). (F) Normalized ψ for 47 of the 130 significantly regulated exons showing the least variation in ψ across unaffected FC (see STAR Methods). Individuals are sorted by splicing dysregulation score (see STAR Methods), also shown above in bars. (G) Heatmap showing enrichment of motifs around the 101 significantly regulated skipped exons relative to all other measured skipped exons. The columns denote the intronic region from +1 to +250 and −250 to −1 of the upstream intron, the skipped exon, and the intronic region from +1 to +250 and −250 to −1 of the downstream intron. (H) Total splicing dysregulation in all human and MBNL KO mouse samples considered as computed using 74 orthologous exons significantly dysregulated (|Δψ| > 0.1, p < 0.05 by rank-sum test) in DM1 patients and MBNL DKO mice.

We performed RNA-seq using RNA from post-mortem FC (Brodmann area 10) of 21 DM1, 4 DM2, and 8 unaffected age- and sex-matched individuals (Figure 1A; see STAR Methods). All libraries satisfied typical quality metrics in FastQC (Andrews, 2010) and were sequenced to a depth of at least 88 million reads to provide sufficient coverage for analyses of gene expression and alternative splicing. The percentage spliced in (ψ) values were estimated by Mixture of Isoforms (MISO) (Katz et al., 2010), and using a threshold of at least 20% change in mean ψ (p < 0.01, rank-sum test), 130 exons were identified to be differentially included between DM1 and unaffected individuals (Figures 1B and 1C; Table S2). These consisted of exons in genes encoding key synaptic scaffolding proteins, cytoskeletal organization components, ion channels, and neurotransmitter receptors, including some previously identified (MBNL2 exon 5, KCNMA1 exon 34, MAPT exon 3, and CSNK1D exon 9) plus additional candidates (GRIP1 exon 21, GABRG2 exon 10, DLGAP1 exon 20, and PALM exon 8). Some of these exons showed greater variability in ψ among DM1 patients relative to unaffected individuals. A similar observation in muscle likely reflects differences in disease severity across patients, partly determined by the extent of MBNL sequestration (Wagner et al., 2016). To assess whether a similar phenomenon might exist in the CNS, we correlated ψ between pairs of exons across all unaffected and DM1 individuals (Figure 1D). ψ for MBNL2 exon 5 correlated strongly with ψ for GABRG2 exon 10 (Pearson R = −0.89), and ψ for GRIP1 exon 21 correlated strongly with ψ for DLGAP1 exon 20 (Pearson R = 0.72). The distribution of R values for all pairwise comparisons of all 130 mis-spliced exons was strongly shifted to the right compared with a null distribution computed following shuffling of sample labels (Figure 1E). These observations suggest that similar to peripheral tissues, mis-splicing in the CNS may exhibit a spectrum of disease severity driven by a common upstream factor.

By visualizing a panel of 47 DM1-affected exons showing minimal variability among unaffected individuals, we observed that some events showed moderate dysregulation among some individuals, whereas others showed more widespread dysregulation, suggesting that some exons are more responsive to disease state than others (Figure 1F). To more precisely quantitate overall mis-splicing, we computed a splicing dysregulation score (mean |Δψ|) (see STAR Methods), as performed similarly in peripheral tissues (Figure 1F) (Wagner et al., 2016). This score did not correlate with age or sex (Figure S1) and may be considered a proxy for overall molecular perturbation. It remains to be seen in future studies whether this score correlates with disease symptoms.

### Mis-spliced exons in DM1 show MBNL motif signatures and suggest intermediate MBNL depletion

To identify *cis*-elements that may be associated with aberrant mis-splicing, we enumerated 5-mers in the regions flanking and within all mis-spliced skipped exons and compared with a set of CNS-expressed control skipped exons (see STAR Methods). We observed a strong signature for MBNL motif (YGCY) enrichment, including TGCTT and GCTGC (Figure 1G) (Lambert et al., 2014; Wang et al., 2012). The pattern of enrichment also reflected expected functional binding patterns of MBNL; i.e., aberrantly included exons showed enriched binding sites within the upstream intron and aberrantly excluded exons showed enriched binding sites within the downstream intron. These observations suggest that similar to the periphery, MBNL sequestration is a major driver of mis-splicing in the DM1 CNS.

Several mouse models have been developed to study loss of MBNL function, including mice globally lacking MBNL2 (MBNL2 knockouts [KOs]) (Charizanis et al., 2012) and mice lacking MBNL1 constitutively and MBNL2 in neurons (MBNL double knockouts [DKOs]) (Lee et al., 2013; Goodwin et al., 2015). To estimate the extent to which MBNL proteins are functionally depleted by expanded CUG repeats in these post-mortem samples, we calculated splicing dysregulation scores for wild-type (WT) mice, MBNL KO mice, and our post-mortem samples using a set of orthologous exons significantly mis-spliced in MBNL1/2 DKO mice and patients (Table S3; see STAR Methods). All patients except for one mild case showed splicing dysregulation between that observed in MBNL2 KO and MBNL1/2 DKO (Figure 1H). In FC, at the RNA level, MBNL2 is ~2-fold more highly expressed than MBNL1, and if one assumed similar protein translation for each MBNL, most individuals studied in this cohort showed at least 66% depletion of MBNL.

### All assessed patients show CNS alleles with >1,000 CTGs, and repeat lengths correlate with splicing dysregulation

**Figure 2.**
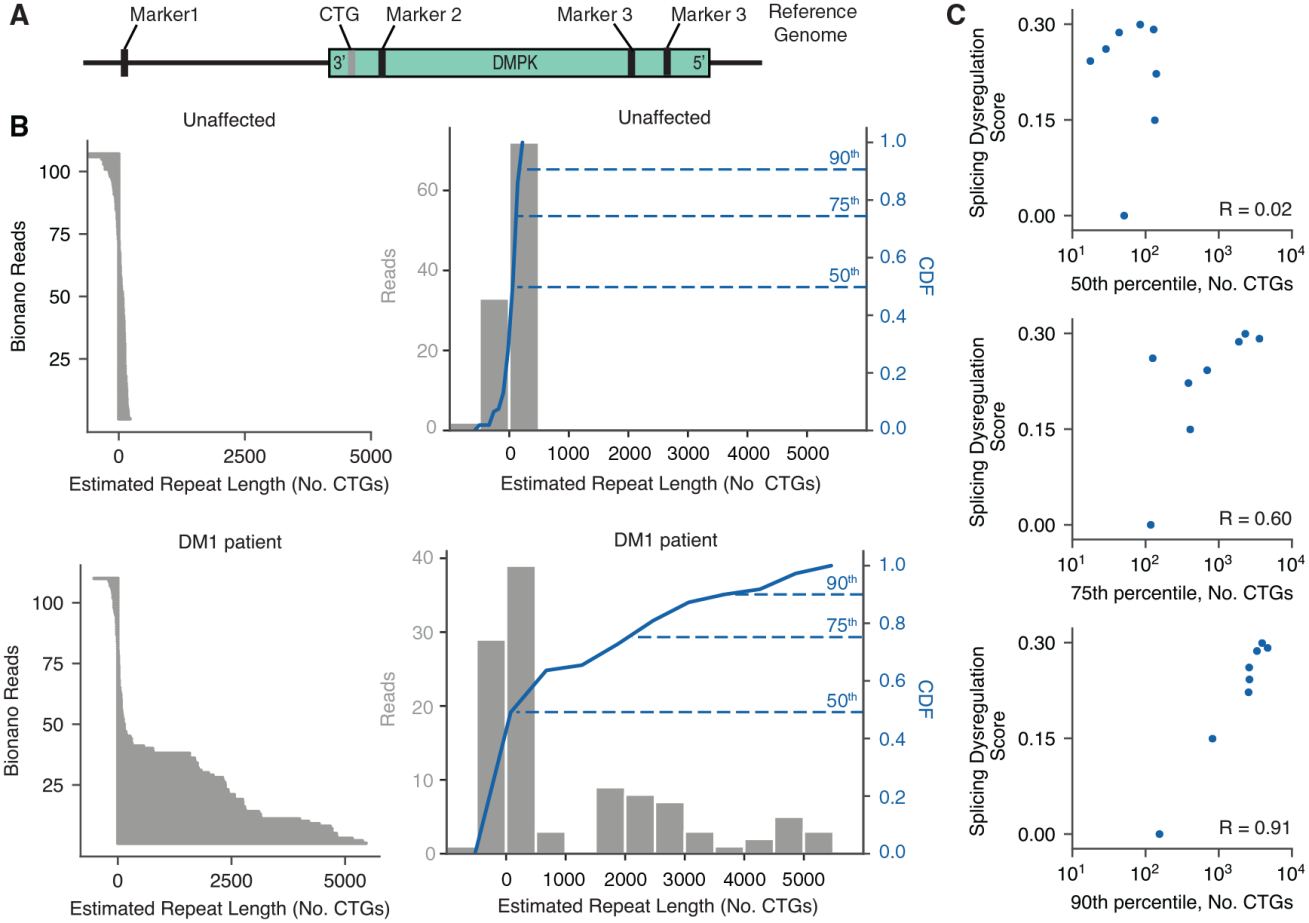
DM1 FC samples show large expansions, and the proportion of long expansions correlates with overall splicing dysregulation (A) DNA fragments from unaffected and DM1 FC samples were labeled by direct labeling enzyme (DLE) and subjected to optical mapping of the DMPK locus. (B) Observed Bionano reads are shown on the left for one unaffected individual (top) and one DM1 individual (bottom). Histograms of the estimated CTG repeat lengths are shown on the right (gray bars) for individuals, along with their cumulative distribution functions (CDFs, blue line). The 50th, 75th, and 90th percentiles of repeat lengths are indicated. (C) Scatterplot of the 50th, 75th, and 90th percentiles of repeat lengths versus total splicing dysregulation across all unaffected and DM1 individuals for which repeat lengths were measured. Pearson correlation values are shown.

To precisely measure repeat lengths at single-molecule resolution, we used an optical mapping technique in which specific restriction sites are fluorescently labeled across the genome and imaged within DNA nanochannels (Barseghyan et al., 2017). We used specific labels near the DMPK locus to estimate CTG repeat lengths, in which 1 micron spans approximately 2 kilobases (Figure 2A; see STAR Methods). We applied this approach to one unaffected control and 7 DM1 FC samples. Although the number of CTG repeat units in the unaffected control was estimated to be ~23 ± 142 SD, a typical DM1 patient showed estimated repeat lengths reaching ~5,000 CTGs. The distribution of lengths in the DM1 patients shifted rightward such that the 50th, 75th, and 90th percentiles of repeat lengths were 84, 2,306, and 3,910 CTGs, respectively (Figure 2B; Figure S1B). Every DM1 sample analyzed showed repeat lengths greater than 1,000 CTGs, and 86% of them showed alleles >4,400 CTGs. The 50th, 75th, and 90th percentiles of repeat lengths were correlated to the previously computed splicing dysregulation scores (Figure 2C). The strongest correlation was observed with the 90th percentile of repeat lengths, or the minimum length of the longest ~20% of all expansion-carrying alleles.

### Alternative splicing of the kinesin binding domain in GRIP1 is perturbed in DM1 and modulates association with KIF5A

**Figure 3.**
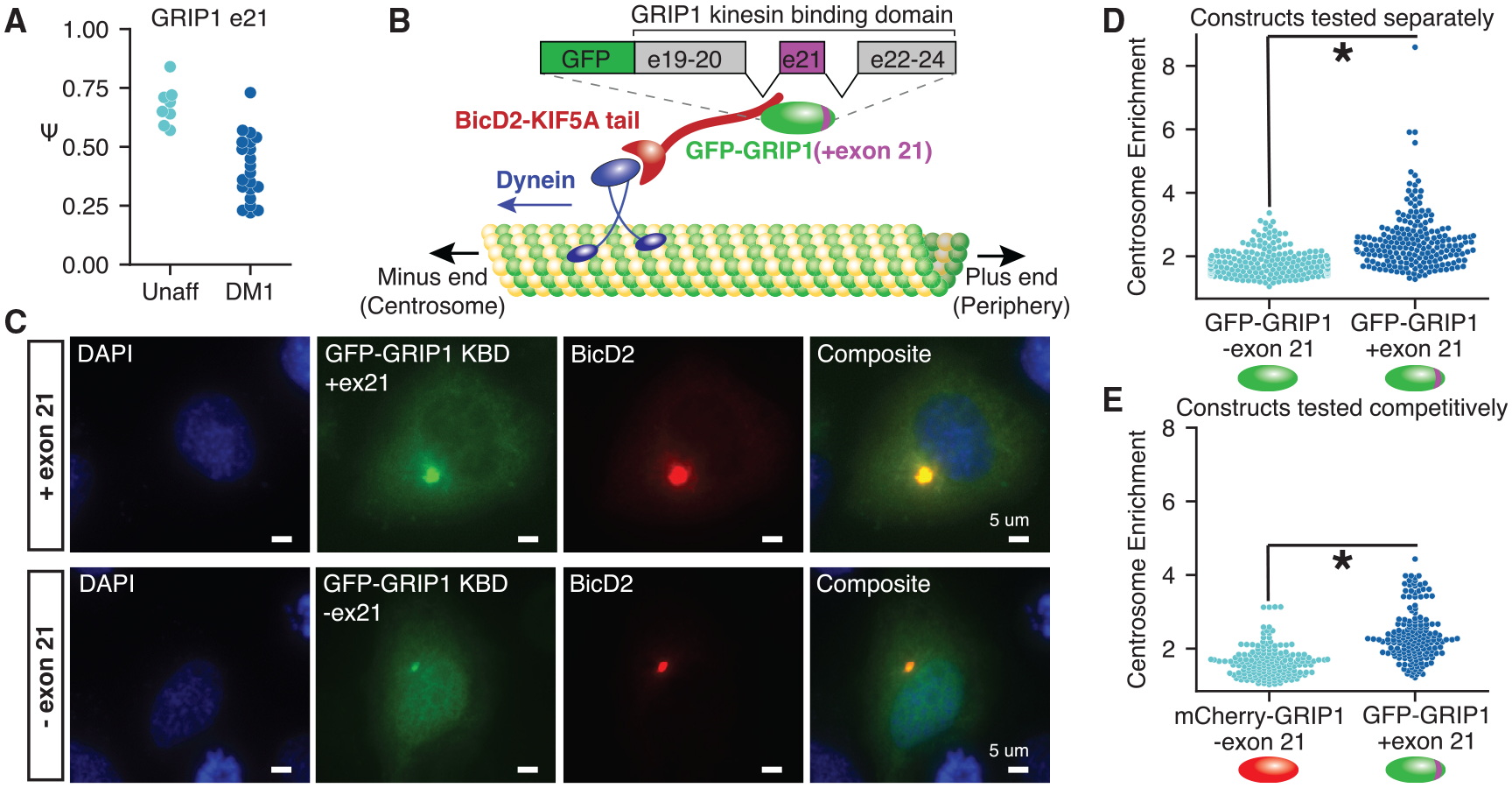
Mis-splicing of GRIP1 in DM1 may lead to changes in kinesin association (A) GRIP1 exon 21 is significantly mis-spliced in DM1 FC. (B) Schematic of GRIP1 kinesin binding domain (KBD) and the centrosome recruitment assay. The C-terminal tail of KIF5A is used as a bait to recruit GFP-GRIP1 fusions to the centrosome. (C) Representative images of the centrosome recruitment assay using GRIP1 KBD fluorescent fusion proteins, with and without exon 21, taken at 403 magnification. (D and E) Quantitation of recruitment efficiency (mean signal at the centrosome divided by mean cytoplasmic signal outside the centrosome) for each construct transfected either (D) independently or (E) competitively, in which the construct with exon 21 was fused to GFP and the construct without exon 21 was fused to mCherry. Significance is shown by an asterisk, and p < 0.01 is shown by a rank-sum test. Cells were quantitated from at least 3 independent transfections.

As shown in Figure 1, multiple synaptic scaffolding proteins contain exons dysregulated in DM1. We focused on exon 21 of GRIP1 (Figure 3A), a gene that plays important roles in localizing AMPA receptors to the synapse and regulating synaptic scaffolding through its 7 post-synaptic density (PDZ) domains. Exon 21 lies within the kinesin binding domain (KBD), which was previously shown to mediate interactions with KIF5A, KIF5B, and KIF5C (Setou et al., 2002). We therefore hypothesized that this exon might modulate the behavior of the KBD. To measure the association of GRIP1 isoforms with KIF5A in cell culture, we generated fluorescent protein fusion constructs to study the behavior of the GRIP1 KBD with and without exon 21. We modified a previously developed centrosome recruitment assay (Bentley and Banker, 2015) and co-transfected these constructs with a BicD2-KIF5A tail, FLAG-tagged construct into Neuro2A cells; BicD2 is a dynein adaptor that directs the KIF5A C-terminal tail to the centrosome, along with its putative cargoes (Figure 3B; see STAR Methods). Association of FP-GRIP1 KBD with the KIF5A tail was quantified by measuring mean intensity at the centrosome relative to mean cytoplasmic intensity. GRIP1 KBD containing exon 21 exhibited stronger centrosome recruitment compared with GRIP1 KBD lacking exon 21 (Figure 3C; see STAR Methods). This increased association with KIF5A was quantitated in experiments in which GFP constructs with and without exon 21 were tested separately (Figure 3D) or competitively, in which mCherry and GFP fusions for each isoform were co-transfected (Figure 3E). In both cases, the GRIP1 KBD with exon 21 was more strongly recruited to the centrosome (p < 1e–25 for single transfections and p < 1e–26 for co-transfections), suggesting an important role for this exon in regulating KIF associations and synaptic recruitment of cargoes.

### Splicing dysregulation in DM1 is largely tissue specific

**Figure 4.**
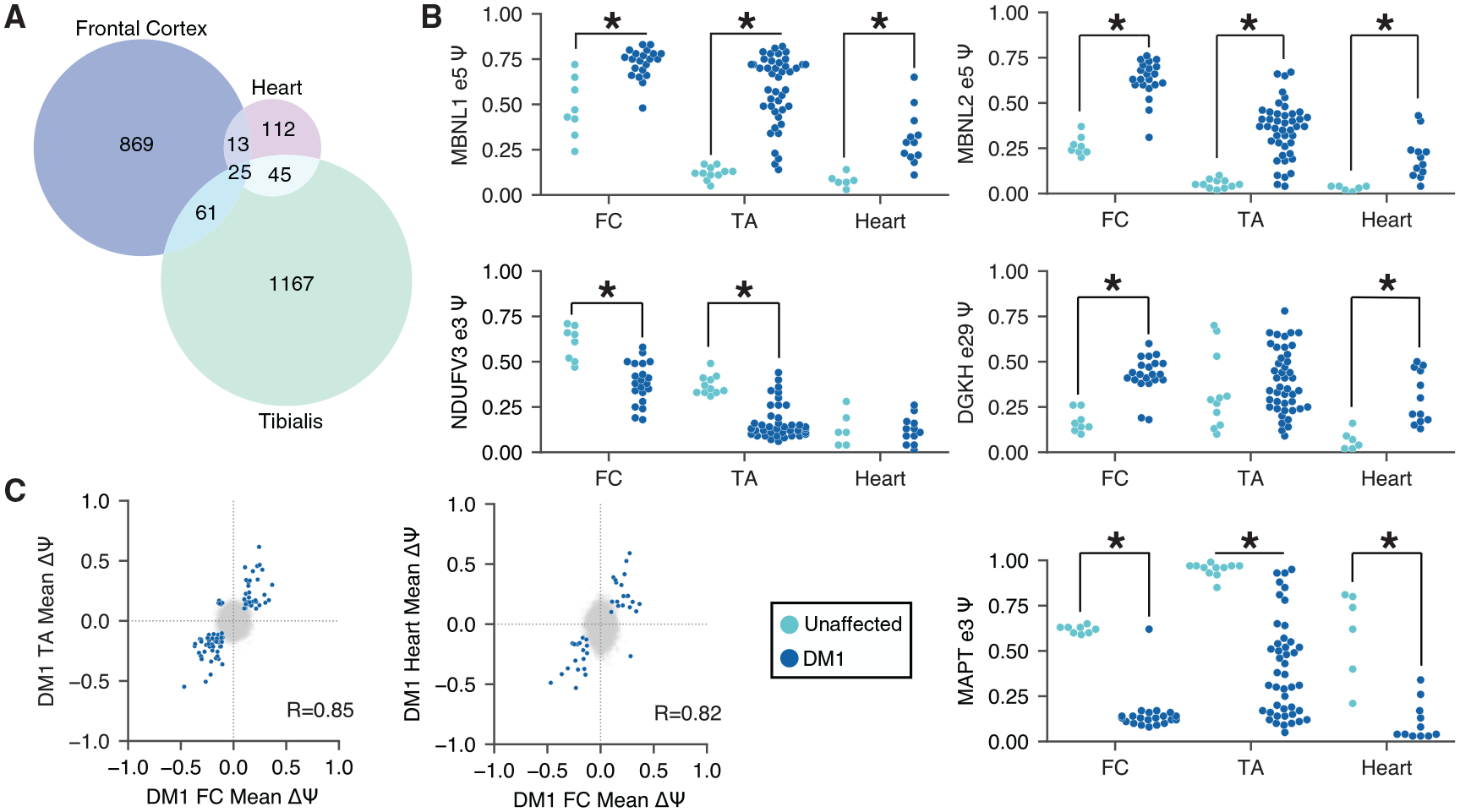
Across DM1 FC, skeletal muscle, and heart, most splicing events are tissue specific (A) Venn diagram showing exons mis-spliced in 1, 2, or all 3 DM1 tissues. (B) ψ for specific exons in FC, skeletal muscle, and heart. Exons significantly mis-regulated in any given tissue are indicated with an asterisk. (C) Scatterplot of ψ for each pair of tissues analyzed. Exons that are significantly regulated (|Δψ| > 0.1, p < 0.01 by rank-sum test) in both tissues are highlighted in blue; 86 are shared between FC and skeletal muscle, and 38 are shared between FC and heart. Pearson correlations for blue points are shown.

To explore potential overlaps in DM1 splicing dysregulation between FC and peripheral tissues, we analyzed previously published RNA-seq data from tibialis anterior (TA) and heart (Wang et al., 2019). We identified exons mis-spliced in each tissue (|Δψ| ≥ 0.1, rank-sum p ≤ 0.01) and assessed 2- and 3-way overlaps. We chose a slightly looser threshold for this analysis compared with what was performed in Figure 1, because the goal was to identify as many exons as possible whose dysregulation might be shared across tissues. Interestingly, at this threshold, only ~10% of the exons dysregulated in FC were also dysregulated in peripheral tissues; ~20% are simply not expressed in peripheral tissues, whereas ~70% are expressed in peripheral tissues but do not show the same dysregulation (Figure 4A). At a more stringent threshold of |Δψ| ≥ 0.2, ~18% of exons dysregulated in FC were also dysregulated in peripheral tissues (Figure S2A). Overlaps between any given pair of tissues revealed many dozens of shared dysregulated exons. Some examples include NDUFV3 exon 3 (significantly regulated only in TA and FC) and DGKH exon 29 (significantly regulated only in heart and FC). The overlap in shared dysregulated exons among all three tissues was 25 shared exons, including MBNL1 exon 5, MBNL2 exon 5, and MAPT exon 3. Although the number of total patients considered within each tissue can influence the total number of exons identified to be significantly dysregulated in any given tissue, we performed sub-sampling analyses to match patient group sizes and found similar proportions of overlap (Figures S2B–S2F).

Among the events that are shared between any pair of tissues, the direction of dysregulation was found to be conserved (Figure 4C), as reflected by the strong correlations in Δψ between TA versus FC (Pearson R = 0.85) and heart versus FC (Pearson R = 0.82). These observations suggest similar mechanisms for splicing dysregulation across all tissues.

### Splicing alterations in DM2 are largely distinct but implicate secondary binding motifs for MBNL and RBFOX

**Figure 5.**
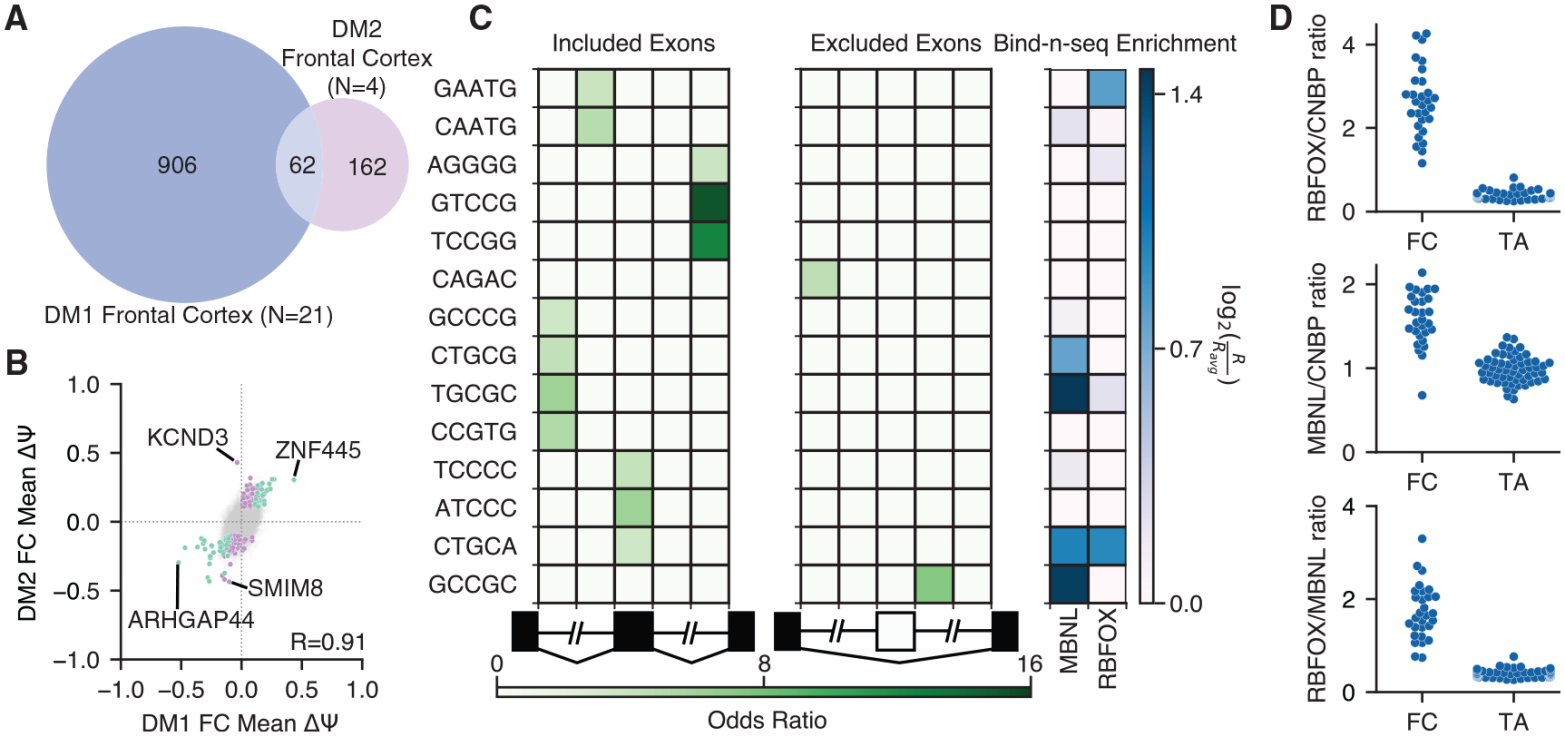
The extent of mis-splicing shared between DM1 and DM2 FC is limited (A) Venn diagram showing exons mis-spliced (|Δψ| > 0.1, p < 0.01 by rank-sum test) in DM1 or DM2 FC or both. (B) Scatterplot of Δψ for DM2 versus DM1. 62 exons identified to be significantly regulated in both DM1 and DM2 (|Δψ| > 0.1, p < 0.01 by rank-sum test) are highlighted in teal, and 162 exons identified to be significantly regulated uniquely in DM2 (|Δψ| > 0.1, p < 0.01 by rank-sum test) are highlighted in purple. DM1-specific events have been omitted for clarity. Pearson correlation for shared (teal) events is shown. (C) Heatmap showing enrichment of motifs around 35 DM2-regulated skipped exons relative to all other measured skipped exons. The columns denote the intronic region from +1 to +250 and −250 to −1 of the upstream intron, the skipped exon, and the intronic region +1 to +250 and −250 to −1 of the downstream intron. Bind-N-Seq enrichment values for MBNL and RBFOX are also shown; enrichments were derived from experiments using 1,080 nM MBNL1 or 1,100 nM RBFOX2. (D) Transcript per million (TPM) ratios of total RBFOX (RBFOX1, RBFOX2, and RBFOX3) versus CNBP, total MBNL (MBNL1, MBNL2, and MBNL3) versus CNBP, and total RBFOX versus total MBNL are shown across FC and skeletal muscle. Note the high concentration of RBFOX in FC relative to skeletal muscle.

DM1 and DM2 are caused by related repeat expansions (CTG and CCTG, respectively). They exhibit elements of shared CNS pathology, including problems with executive function and hypersomnolence, and potential elements of shared molecular pathology. Both diseases are thought to be driven by MBNL sequestration, but RBFOX may also play a role in DM2 (Sellier et al., 2018). We profiled four DM2 FC samples, because there was limited availability of post-mortem tissue. Consistent with previous observations in muscle and blood cells, intron 1 of CNBP was retained in all DM2 samples but was efficiently spliced in all DM1 and unaffected samples (Figure S3A) (Sznajder et al., 2018; Wang et al., 2019). We compared mis-splicing in DM2 to that observed in DM1 and identified exons exclusively regulated in one disease or shared by both (|Δψ| ≥ 0.1, rank-sum p ≤ 0.01) (Table S4). Approximately ~28% of exons dysregulated in DM2 were also dysregulated in DM1 (Figure 5A) and generally changed in the same direction (Figure 5B, shown in teal). At a threshold of |Δψ| ≥ 0.1, ~22% of exons dysregulated in DM2 were also dysregulated in DM1 (Figure S2B). Many exons unique to DM2 (Figure 5B, shown in purple) trended toward regulation in DM1 but did not reach significance. To estimate the extent to which our sample size for DM2 might limit the power to detect dysregulated exons in DM2, we repeatedly sub-sampled 4 of 21 DM1 samples and re-estimated the overlap between DM2-and DM1-regulated events (Figures S3C–S3G). We estimate that with the 4 DM2 samples analyzed, we capture 25%–50% of the repertoire of altered splicing that may exist in DM2 (see STAR Methods).

To identify *cis*-elements that may play a role in regulating DM2-specific mis-splicing events, we enumerated 5-mers in regions flanking and within all significantly regulated skipped exons and compared these with a set of CNS-expressed control skipped exons (see STAR Methods). In contrast to analyses of exons differentially regulated in DM1, enrichment of canonical MBNL and RBFOX motifs was not readily apparent (Figure 5C, left panel). The absence of canonical YGCY and UGCAU/GCAUG motifs for MBNL and RBFOX, respectively, motivated us to examine the expression levels of MBNL, RBFOX, and CNBP genes in TA and FC to assess whether the stoichiometry of each player might provide additional insights (Figure 5D). In all comparisons, we found that the ratio of gene expression for MBNL (1, 2, and 3) and/or RBFOX (1, 2, and 3) versus CNBP was higher in FC relative to TA, suggesting a greater buffering capacity (i.e., resistance to depletion) in FC. In contrast, the ratio of DMPK to MBNL is more similar in FC relative to TA (Figure S3H), consistent with similar signatures of MBNL motif enrichment around exons mis-spliced in DM1 FC and TA (Wang et al., 2019).

Analyses of *in vitro* binding preferences for RBPs indicate that the concentration of the RBP can influence the extent to which optimal versus sub-optimal motifs are bound (Lambert et al., 2014; Begg et al., 2020). That is, the best motifs are bound when the RBP concentration is limiting, but sub-optimal motifs are also bound when the RBP concentration is high. Indeed, upon analyzing Bind-N-Seq data for MBNL1 and RBFOX2 at elevated protein concentrations (1,080 nM MBNL1 and 1,100 nM RBFOX2) (Lambert et al., 2014), we observed positive binding enrichments for both MBNL and RBFOX among DM2-enriched motifs (Figure 5C, right panel). We also assessed whether exons exclusively regulated in DM2, but not in DM1, showed enrichment for sub-optimal RBFOX binding sites, but we could not detect effects reaching statistical significance (data not shown).

### Gene expression changes suggest neuroinflammation, are cell-type specific, correlate with splicing dysregulation, and reveal potential biomarkers

**Figure 6.**
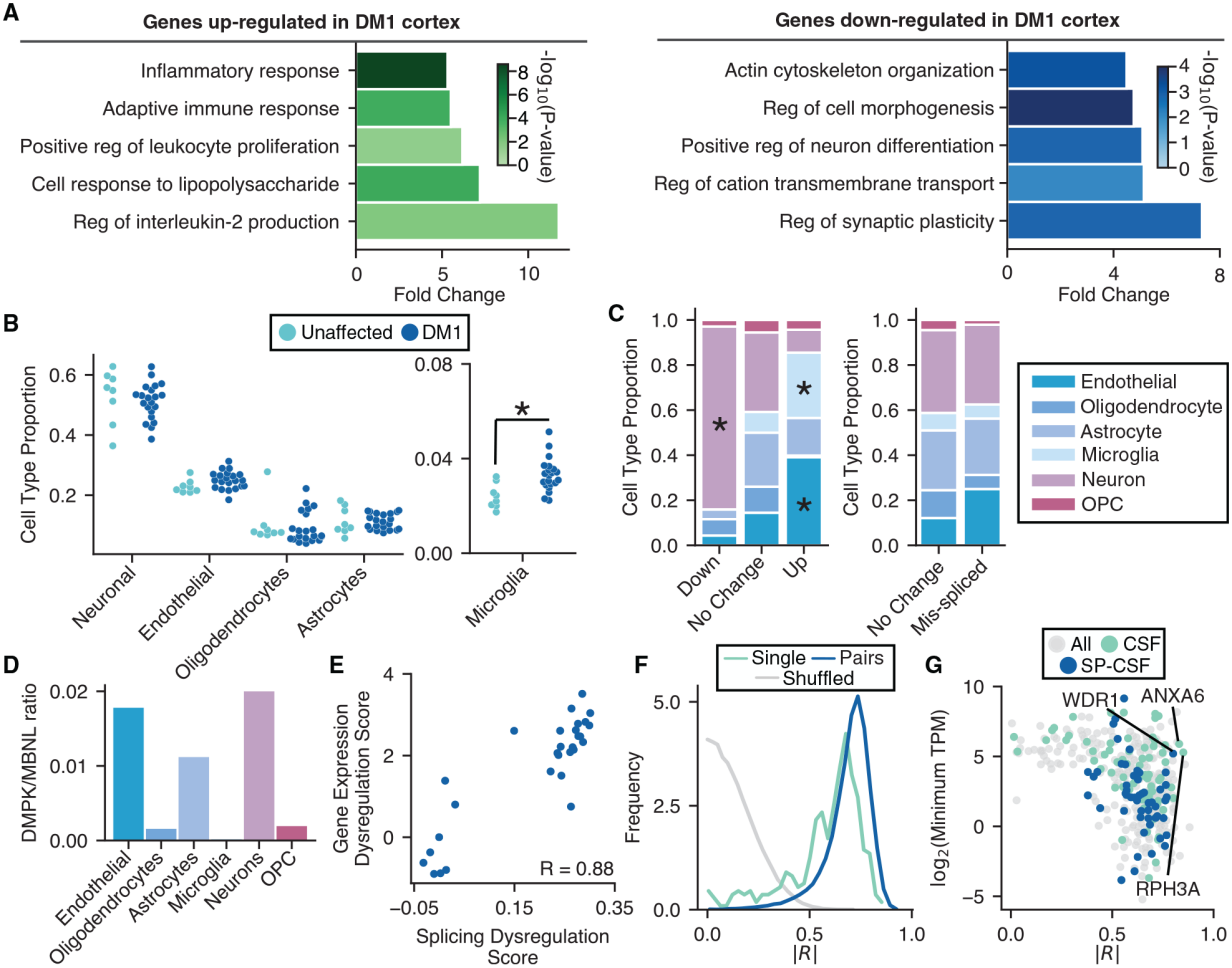
Analysis of gene expression changes reveals neuroinflammation and potential biomarkers (A) Gene Ontology analysis of genes upregulated (green) and downregulated (blue) in DM1 versus unaffected FC (top five categories shown for each, selected by fold change). (B) Proportions of neurons, microglia, endothelial cells, oligodendrocytes, and astrocytes in each FC sample were estimated by Bayesian inference using published transcriptome profiles (see STAR Methods) and plotted. Significance is shown by an asterisk. (C) Proportion of up-, non-, and downregulated genes derived from specific cell types is shown (left panel). The proportion of mis-spliced and unaffected genes derived from specific cell types is also shown (right panel). Cell-type specificity for genes was determined using publicly available single-cell sequencing data (see STAR Methods). Significance is shown by an asterisk. (D) TPM ratios for DMPK versus total MBNL (MBNL1, MBNL2, and MBNL3) across various CNS cell types. (E) Scatterplot of splicing dysregulation score versus gene expression dysregulation score (see STAR Methods). Pearson correlation is shown (p = 3e–10). (F) Pearson correlations between splicing dysregulation score and log_2_(TPM) for each dysregulated gene were computed and plotted as a histogram (teal). Samples were shuffled and correlations were recomputed and plotted (gray). A similar score was also computed between splicing dysregulation and all pairs of genes (blue) (see STAR Methods). Absolute values for all correlations were used for plotting. Similarities of distributions were assessed by a KS test; p < 1e–300 when comparing shuffled to single genes, and p < −200 when comparing shuffled to pairs of genes. (G) Scatterplot of the single-gene correlations computed in (F) versus log_2_(TPM) for those genes. Genes encoding proteins detectable in CSF, and those additionally found to have signal sequences (SignalP) (see STAR Methods) are highlighted in teal and blue, respectively.

Using Kallisto (Bray et al., 2016) and Sleuth (Pimentel et al., 2017), we estimated gene expression changes (Table S5 and Table S6) for all DM1, DM2, and unaffected FC samples. We found that 235 genes were significantly upregulated (q < 0.01 and |fold-change| ≥ 1) in DM1 FC and 145 genes were significantly downregulated relative to unaffected FC. Gene Ontology analyses (Figure 6A) showed that upregulated genes were enriched in adaptive immune response, cell regulation of leukocyte proliferation, and inflammatory response. Downregulated genes were enriched in actin cytoskeleton organization, regulation of cation transmembrane transport, and regulation of synaptic plasticity. Because FC contains various cell types, we investigated whether there might be changes in cell-type composition between patients and unaffected controls. Using published single-cell RNA-seq data (Darmanis et al., 2015), we built a Bayesian inference approach to estimate the proportion of neurons, endothelial cells, astrocytes, oligodendrocytes, microglia, and oligodendrocyte precursor cells within each sample. This model was able to detect frank neuronal cell loss in the context of Alzheimer’s disease (van Rooij et al., 2019) (Figure S4) but did not show neuronal cell loss in DM1 (Figure 6B). However, we did observe a signature for increased microglial composition among patients, consistent with an inflammatory response (Figure 6B) and gliosis that has been previously observed (Itoh et al., 2010). We classified up- and downregulated genes by their cell type of origin (genes expressed similarly across multiple cell types were omitted from this analysis; see STAR Methods) and observed a strong signature for neuronal genes being downregulated, as well as a strong signature for endothelial and microglial genes being upregulated (Figure 6C, left panel). In contrast, splicing changes were inferred to be present in all cell types studied (Figure 6C, right panel). The ratio of DMPK to MBNL (1, 2, and 3) was highest in endothelial cells, astrocytes, and neurons, perhaps providing conditions most conducive to strong MBNL sequestration in those cell types (Figure 6D).

We next investigated whether gene expression changes correlate with splicing dysregulation across DM1 and unaffected controls. We developed a gene expression dysregulation score, which summarized the total change in gene expression among up- and downregulated genes (see STAR Methods). These scores were then correlated with previously calculated splicing dysregulation scores, revealing a strong correlation (Pearson R = 0.88) (Figure 6E). To investigate whether a smaller subset of genes could be as informative as assessment of all dysregulated genes, we correlated individual and pairs of gene expression changes to splicing (Figure 6F) and compared these with correlations of shuffled data. Many gene expression changes showed correlations >0.8 and may be strong candidates for markers of pathology. For purposes of clinical studies or trials, however, a marker would ideally be accessible via CSF. We overlapped our FC gene expression changes with proteomics studies of CSF (Macron et al., 2018) and identified genes that (1) encode proteins detectable in CSF, with or without canonical signal sequences, determined by SignalP (Nielsen et al., 1997); (2) show strong correlations with splicing dysregulation; and (3) are expressed at reasonable levels in the CNS to maximize sensitivity of detection (Figure 6G). Several candidates are highlighted; WDR1 is upregulated in DM1 FC, and ANXA6 and RPH3A are downregulated. WDR1 is involved in disassembly of actin filaments and cell migration (Kato et al., 2008), ANXA6 is involved in vesicle fusion (Lin et al., 1992), and RPH3A plays essential roles in synaptic vesicle release (Burns et al., 1998). These gene expression changes may also be reflected at the RNA or protein level in DM CSF, providing an accessible route by which to measure CNS disease status in future studies. Their utility as biomarkers remains to be validated.

## DISCUSSION

In this study, we have generated and characterized transcriptomes derived from DM1 and DM2 FC and associated controls. We identified a high-confidence set of splicing events whose dysregulation correlates with CTG repeat length as measured by optical mapping, an amplification-free approach to sizing repeats at a single-molecule level. Although previous analyses of progenitor allele lengths have observed correlations to age of onset and disease severity (Cumming et al., 2019), a correlation between actual repeat lengths and extent of mis-splicing in the same tissue has never been measured. Although we did not have phenotypic information about individuals, these observations suggest that the extent of mis-splicing and repeat length may also underlie the severity of disease symptoms. Alterations in the splicing patterns of many neurotransmitter receptors and synaptic scaffolding proteins were identified, revealing candidates that may play important roles in the neurobiology of DM1 symptoms. Notably, mis-splicing of some exons tended to enhance the activity of inhibitory machinery and dampen the excitatory machinery. For example, skipping of GABRG2 exon 9 is associated with increased sleep times in mice treated with benzodiazepines (Homanics et al., 1997), and skipping of exon 8 of PALM, a developmentally regulated exon, is associated with reduced filopodia formation, dendritic spine maturation, and AMPA receptor recruitment (Arstikaitis et al., 2008). Exon skipping within the KBD of GRIP1 (Setou et al., 2002), an important synaptic adaptor for AMPA receptors, may decrease the efficiency by which AMPA receptors are recruited and stabilized at synapses.

Analyses of *cis*-elements flanking dysregulated exons revealed that MBNL-bound motifs were the predominant signature, suggesting that functional depletion of MBNL is a major driver of splicing changes in DM1 FC. Consistent with these observations, orthologous alternative exons in MBNL2 KO and MBNL1/2 DKO mice showed changes in splicing similar to DM1, with the severity of mis-splicing in virtually all DM1 patients showing intermediate depletion, e.g., between MBNL2 KO and MBNL1/2 DKO. When considered with estimates of repeat lengths we obtained using optical mapping, these observations suggest that CTG tracts of more than 1,000 repeat units, and perhaps up to 4,000, are required to achieve this level of sequestration. This could explain why some animal- and cell-based DM1 models do not recapitulate the full extent of mis-splicing observed in human tissue. Correlations between mis-splicing and repeat lengths were strongest when considering the longest 20% of disease alleles (the longest 10% of all alleles), suggesting that somatic instability plays a critical role in disease onset and severity and that potentially, a subset of cells is responsible for the severest molecular signatures observed. The expansion process may take decades, consistent with some DM1 symptoms only manifesting in adult or late-adult life. Given that full depletion of MBNL leads to nuclear export of expanded DMPK (Zu et al., 2017), it is possible that cells with extreme repeat lengths contain CUG-derived repeat-associated non-AUG translation peptides, invoking additional pathological mechanisms. Future work should focus on which cell types harbor these extremely large alleles and the extent to which those cells contribute to the bulk transcriptome signatures we observe here. Although we have focused on FC in this study, the clinical presentation of DM and imaging studies indicate abnormalities in multiple brain regions. Future studies should similarly examine transcriptome changes and repeat length distributions in those regions so that molecular features can be better linked to particular symptoms. Of particular interest may be the hippocampus and amygdala, because these regions show anatomical differences in DM1 and are known to play roles in memory processing and emotional experiences (van der Plas et al., 2019).

Surprisingly few transcripts showed mis-splicing across DM1 TA, heart, and FC. Even when exons are expressed in all 3 tissues, many show mis-splicing in only 2 of 3 tissues. Tissue-specific regulation of RBPs or *trans*-factor environments may be responsible for these differences. Although a greater number of DM2 FC samples will be needed to fully characterize transcriptome changes in DM2, we found limited overlap in mis-splicing between DM1 and DM2. Although shared events were generally dysregulated in the same direction in both DM1 and DM2, there was also a substantial subset of events dysregulated in only DM1 or only DM2. These observations may differ from those in peripheral tissues, but gene expression levels of RBFOX family members relative to MBNL or CNBP are greater in the CNS compared with skeletal muscle. RBFOX proteins have been proposed to buffer MBNL sequestration (Sellier et al., 2018), and this phenomenon may play a more substantial role in the CNS, in which RBFOX proteins may be in greater stoichiometric excess than in muscle. Finally, differences in cell-type-specific expression of DMPK, CNBP, MBNL, and RBFOX may also play a role in producing distinct transcriptome signatures of each disease subtype.

By implementing a Bayesian inference approach to infer cell-type composition using published single-cell RNA-seq data, we found no significant neuronal cell loss in the FC samples we studied. However, there was a significant increase in microglia. Downregulated genes tended to be those expressed preferentially in neurons, and upregulated genes tended to be those expressed preferentially in endothelial and microglial cells. These observations suggest a neuroinflammatory response accompanied by neuronal dysfunction, but not overt neurodegeneration, at least in this brain region. The complexity of cell-type composition in the CNS concurrent with somatic instability prompts future studies to directly assess variability of transcriptome alterations at a single-cell level to separate those cells driving disease versus those responding to paracrine signals. Overall, gene expression changes correlate strongly with mis-splicing, providing an opportunity to develop gene expression or protein-based biomarkers of disease severity. Indeed, overlapping observed gene expression changes with published CSF proteomics datasets suggest strong candidates for further evaluation. RPH3A plays roles at both the pre- and the post-synapse; it interacts with RAB3A (Shirataki et al., 1993) and SNAP25 (Ferrer-Orta et al., 2017) to regulate synaptic vesicle trafficking at the pre-synapses and interacts with PSD-95 to stabilize NMDA receptors at the post-synapse (Stanic et al., 2015). Related to this pathway, RAB3A protein has been observed to be elevated in a CNS mouse model of DM, as well as in DM1 FC (Hernández-Hernández et al., 2013). Finally, RPH3A has been observed to be downregulated in HD patients (Smith et al., 2007). Therefore, these candidates may also inform us about pathways perturbed specifically in DM or broadly across neurological diseases.

## STAR★METHODS

### RESOURCE AVAILABILITY

#### Lead Contact

Further information and requests for resources and reagents should be directed to and will be fulfilled by the lead contact, Eric T. Wang (eric.t.wang@ufl.edu).

#### Materials Availability

Plasmids generated for this study are available from the lead contact without any restriction.

#### Data and Code Availability

All RNA-seq data generated in this study are available in GEO (accession number GSE157428). The code generated during this study is available at https://github.com/brittneyotero/dm1-frontalcortex

### EXPERIMENTAL MODEL AND SUBJECT DETAILS

#### Cell lines

Mouse Neuro2a cells were used in this study, grown at 37°C and 5% CO_2_ in DMEM media containing 10% fetal bovine serum.

#### Human subjects

Adult frontal cortex samples were obtained from post-mortem frozen brains, totalling in 21 DM1 samples, 4 DM2 samples and 8 unaffected controls. Samples ranged in age from 39 to 83 and were split across both sexes. Details about human samples used can be found in Table S1. Appropriate IRB approval was obtained at institutions to allow for post-mortem collection and analysis of tissue.

### METHOD DETAILS

#### Tissue and RNA collection, RNA-seq library construction

Autopsy samples were obtained from Stanford University, The Research Resource Network Japan, University of Rochester Medical Center and the NIH Biobank. Tissue samples were homogenized in TRIzol using the Omni bead ruptor followed by the Direct-zol RNA Miniprep kit, with DNase I treatment. RNA quality and abundance were assessed using fragment analysis (Agilent/Advanced Analytical). Samples with an RQN > 4 were further processed. RNA-seq libraries were constructed using the NEBNext Ultra II Directional RNA library prep kit for Illumina, using ribosomal RNA depletion followed by strand-specific RNA-seq preparation. Samples were amplified with PCR for 9–11 cycles and sequenced using the Illumina NextSeq 500 v2 with 75 nucleotide paired end reads. Samples were sequenced across multiple runs to account for possible batch effects, and reads were pooled so that all samples had at least 88 million reads.

#### Read mapping, gene expression quantitation and isoform quantitation

Upon passing FASTQC metrics for quality control (Andrews, 2010), reads were mapped to the hg19 genome using STAR (Dobin et al., 2013). Gene expression was quantified using Kallisto (Bray et al., 2016), with hg19 Refseq tables as a reference. Differentially regulated genes were identified using Sleuth (Pimentel et al., 2017). Isoform percent spliced in (PSI, Ψ) values were quantified by MISO (Katz et al., 2010) using hg19 v2.0 MISO annotations (http://genes.mit.edu/burgelab/miso/annotations/ver2/miso_annotations_hg19_v2.zip).

#### Identification of dysregulated exons and calculation of splicing dysregulation score

Splicing events were assessed for significant dysregulation across individuals by rank-sum test. One hundred thirty DM1 events and 59 DM2 events were found to meet criteria of |Δψ| > 0.2, p < 0.01 (rank-sum test). To assess a false discovery rate, psi values were shuffled among individuals for each event, and the rank-sum test was performed again. After shuffling, 6 out of 130 events remained significant, so we estimate a false discovery rate of < 5%. Of the 130 significantly regulated exons in the DM1 frontal cortex, those with a psi range < 0.25 among unaffected samples were chosen and shown in the heatmap in Figure 1F. Patients were ordered along the x axis by their splicing dysregulation score. This score was derived from the average magnitude of delta psi across all 130 significantly regulated exons, compared to the mean psi of the unaffected samples. The scores themselves are visualized in the corresponding bar graph above the heatmap.

#### Splicing event cross-correlations

Cross-correlations were computed for psi values across individuals, between every pair of differentially regulated exons. The histogram of Pearson correlation values is shown in Figure 1E in black. To compute a null distribution, psi values were shuffled among individuals, and Pearson correlationswere re-computed between all pairs. The histogram of Pearson correlations for shuffled values isshown in gray.

#### Motif Analysis

Of the 130 significantly regulated splicing events in the DM1 frontal cortex, 101 skipped exon events (31 aberrantly included exons and 70 aberrant excluded exons) were used to analyze motif signatures. Sequences from 5 distinct regions were assessed for each event: 250 bases downstream of the upstream exon, 250 bases upstream of the skipped exon, the skipped exon itself, 250 bases downstream of the skipped exon, and 250 bases upstream of the downstream exon. Four-mers within these regions were enumerated and compared to the same regions around all other skipped exon events observed in our dataset. Significance for 4-mer enrichment was determined using a binomial test with Bonferroni correction. A similar analysis was performed for the top 59 events in DM2 frontal cortex, 35 of which were skipped exon events (17 aberrantly included exons and 18 aberrantly excluded exons). To provide additional resolution with respect to motif sequences, which facilitated comparison to 5-mer Bind-N-Seq data (Lambert et al., 2014), 5mers were enumerated along each sequence from these regions, rather than 4mers.

#### Analysis of exons conserved between human and mouse

Previously published RNA-seq datasets of MBNL2 KO (Charizanis et al., 2012) and MBNL1/2 Nestin-cre DKO (Goodwin et al., 2015) mice were analyzed. MISO was used to calculate PSI values across all captured events and significance was calculated by rank-sum test. Events were filtered by |Δψ| > 0.1, p < 0.05 and intersected with significantly mis-spliced events in the DM1 patients using human-mouse orthologous exons as defined by Ensembl. Seventy-nine exons were found to be dysregulated in both human DM1 patients and MBNL DKO mice, and 39 exons were found to be dysregulated in both human DM1 patients and MBNL2 KO mice. Of the 79 exons in both human DM1 and MBNL DKO, 77 were observed in all 3 datasets and used to calculate a splicing dysregulation score as defined above. Samples were ordered along the x axis according to this score in Figure 1H.

#### Assessment of cell type composition by Bayesian Inference

Single-cell RNA-sequencing of human adult temporal lobe (Darmanis et al., 2015) was analyzed to obtain cell type-specific markers in neurons, endothelial cells, microglia, astrocytes, oligodendrocytes, and oligodendrocyte precursor cells (OPCs). The top 50 enriched genes in each cell type were compiled and of these 300, 220 were expressed in our dataset. Using these cell type markers, a Bayesian inference model was built using Pymc3 (Salvatier et al., 2016) to estimate relative proportions of all cell types across all samples. We estimated the proportion of each cell type by implementing a Bayesian framework in which p(cell type | expression data) f p(expression data | cell type) * p(cell type). The priors for each cell type were initialized as Dirichlets, and the sum of all cell type proportions was constrained to 100%. Reported cell type proportions are derived from the mean of the inferred posterior distributions. The estimated proportion of OPCs was < 2.5% and was eliminated from the model; final cell type proportions are reported using a model that incorporates only neurons, endothelial cells, microglia, astrocytes, and oligodendrocytes and uses 184 cell type marker genes.

#### Assessment of cell type-specific gene expression and splicing changes

Cell type specificity of genes was calculated by dividing the recorded expression of each gene in a given cell type by the expected expression of that gene if that gene were equally expressed across all cell types. We designated genes with scores > 3 as specific. The number of up- and downregulated genes (and genes that do not change) was enumerated. A Fisher exact test was used to determine whether particular cell types showed enrichment of differentially regulated genes versus genes not showing changes. A similar analysis was performed with differentially regulated exons versus all exons captured in the dataset not showing regulation.

#### Gene ontology analysis

Gene Ontology analysis was performed on all significantly up- and downregulated genes (235 and 145 genes, respectively), with all expressed genes in the dataset used as the background, using http://geneontology.org/. Categories were collapsed using Revigo (Supek et al., 2011) and the top 5 categories (sorted by enrichment) are shown.

#### Gene expression dysregulation score, correlations, and overlap with CSF proteomics and SignalP

The gene expression dysregulation score was calculated using the significantly up- and down- regulated genes; it was computed as the average magnitude of logged gene expression changes relative to the mean log_2_(TPM) of all unaffected samples. log_2_(TPM) for all up- and downregulated genes were correlated to the splicing dysregulation score across all individuals (Figure 6F). Pearson correlations were calculated and shown on the histogram in green. Additionally, scores were derived for all possible pairs of genes among significantly up- and downregulated genes; the score for pairs of genes going in the same direction was defined as the sum of log(TPM) and the score for pairs of genes going in opposite directions was defined as the difference of log(TPM). Pearson correlations for these scores relative to splicing dysregulation score were calculated and shown on the histogram in blue. To obtain a null distribution for correlations, expression values for all up- and downregulated genes were shuffled and compared again to splicing dysregulation; Pearson correlations were calculated and shown on the histogram in gray. Regulated genes were intersected with genes detected in CSF by proteomics (Macron et al., 2018) to determine whether any genes would be strong biomarker candidates (Figure 6G), shown in teal. This subset of genes was also scored by SignalP version 5 (Nielsen et al., 1997) to identify signal peptides, shown in blue.

#### Bionano Saphyr sample preparation and mapping

For each sample, 10 mg brain tissue was homogenized, embedded in agarose plugs and incubated with lysis buffer and Proteinase K at 50°C overnight. RNase A was added and incubated for 1 hour at 37°C. Agarose was digested with agarase and the purified DNA was subjected to drop dialysis for 4 hours and quantified by the Qubit dsDNA BR assay. Purified DNA was labeled using the Direct Label Enzyme (DLE) method (Bionano Genomics). A total of 750ng of DNA was labeled using the DLE-1 kit following the manufacturer’s instructions and then treated with proteinase K. The DNA was stained with YOYO-1 according to the DLE-1 kit instructions and homogenized by HulaMixer. The stained sample was incubated overnight at room temperature. Labeled and stained DNA was loaded onto the Bionano Genomics Saphyr chip. DNA was linearized in the nanochannel array by electrophoresis. Strands were imaged and the backbone and labels detected by Bionano image detection software. Single-molecule maps were assembled into consensus maps. Consensus maps were refined and merged based on overlapping segments. The final consensus maps were aligned to the GRCh38 human reference genome. Repeat expansions in the DMPK locus were identified by distances between flanking labels in the single-molecule maps aligned to the region.

#### GRIP1 constructs and cloning

Double-stranded gene fragments encoding for GRIP1 kinesin-binding domain (GRIP1-KBD) +/− exon 21 were synthesized by Integrated DNA Technologies (IDT). The pEGFP-C1 (Clontech) plasmid containing a CMV promoter and C-terminal EGFP was used. Using the InFusion HD Cloning Kit (Clontech), GRIP1-KBD ± exon 21 was inserted into the pEGFP-C1 plasmid backbone between the SalI and BamHI sites. mCherry sequence was obtained from a pcDNA3.1 (Invitrogen) plasmid. An mCherry-C1 plasmid was subsequently constructed through excision and replacement of pEGFP between the AgeI and BglII sites. GRIP1-KBD -exon 21 was then cloned into mCherry-C1 as described above.

#### Centrosome recruitment assay and quantification

Mouse Neuro2a cells (Olmsted et al., 1970) were grown at 37°C in Dulbecco’s Modified Eagle Medium (Cytiva) containing 10% fetal bovine serum, D-glucose, L-glutamine, sodium pyruvate, streptomycin, and penicillin. Cells were trypsinized and plated onto glass chamber slides 1–2 days prior to transfection. To generate the BicD2-KIF5A expression plasmid, first a pcDNA3.1-BicD2-KIF1Balpha plasmid was generated. The pcDNA3.1-V5-His-TOPO backbone was cut with BamHI and EcoRV. BicD2 was amplified from a BicD2-FKBP expression plasmid provided by Gary Banker using primers GCTAGTTAAGCTTGGTACCGAGCTCGGATCCATGGATAT CATGGATTACAAGGATGAC and CCACCCCCTCCCGAACCTCCGCCCCCTCTAGAGACGGTCCGATCT. The KIF1Balpha tail was amplified from mouse cDNA using primers GGAGGTTCGGGAGGGGGTGGCTCAGATACATCCATGGGGTCCCTC and GAGCGGCCGCCACTGTGCTGGATATCCTAGACTGTGGTTTCTCGACCT. The BicD2 PCR product and KIF1Balpha PCR products were joined together by PCR using the outer primers, and cloned into the pcDNA3.1 backbone by InFusion HD cloning (Clontech). The pcDNA3.1-BicD2-KIF1Balpha plasmid was then cut using SanDI and NotI, and the KIF5A tail was amplified from human cDNA using primers GCCTCAGTAAATTTGGAGTTGACTGC and TTAGCTGGCTGCTGTCTCTTGG, followed by GCTCAGATACATC CATGGGGTCCCTCGCCTCAGTAAATTTGGAGT and GGGCCCTCTAGACTCGAGCGGCCGCTTAGCTGGCTGCTGTCTCTT. The PCR product was cloned into the backbone also by InFusion HD cloning to generate the BicD2-KIF5A tail construct. Constructs containing FP-GRIP1-KBD ± exon 21 and FLAG-BicD2-KIF5A were expressed by transfection using TransIT-LT1 (Mirus). Approximately 24 hours after transfection, cells were fixed using 4% paraformaldehyde and permeabilized with 0.2% Triton X-100. Cells were stained with a rabbit monoclonal anti-DYKDDDDK tag antibody (D6W5B; Cell Signaling Technologies) followed by an Alexa Fluor 647-conjugated goat anti-rabbit secondary antibody (Abcam). Cells were then mounted in Fluoroshield (Millipore-Sigma). Cells were imaged using the Zeiss LSM880 microscope by epifluorescence with an AxioCam MRm camera and Apochromat 40x/1.3 NA objective. Tile scans were gathered for each condition using ZEN software (Carl Zeiss International). Image analysis and quantitation of tile scans were performed using ImageJ/Fiji (Schindelin et al., 2012). Intensity values for whole cells and centrosomes were manually traced and quantitated using EGFP or mCherry (whole cell) and FLAG (centrosome) signals as masks. Intensity values from these tracings were used to quantify recruitment of GRIP1-KBD to the centrosome by BicD2-KIF5A, as represented by the ratio of centrosomal signal to whole-cell signal.

### QUANTIFICATION AND STATISTICAL ANALYSIS

Statistical analysis for this study was done using custom python scripts. Tests, thresholds and significance values are detailed in the results, methods and figure legends.

## Supplementary Material

Document S1

Table S1

Table S2

Table S7

Table S6

Table S5

Table S4

Table S3

## Figures and Tables

**Table:** KEY RESOURCES TABLE

REAGENT or RESOURCE	SOURCE	IDENTIFIER
Antibodies		
Rabbit monoclonal anti-DYKDDDDK tag	Cell Signaling Technologies	Cat#D6W5B; RRID:AB_2572291
Alexa Fluor 647-conjugated goat anti-rabbit secondary	Abcam	Cat#ab150079; RRID:AB_2722623
Biological Samples		
DM1 frozen post-mortem frontal cortex samples	Stanford University, The Research Resource Network Japan, University of Rochester Medical Center	N/A
Unaffected frozen post-mortem frontal cortex samples	NIH Biobank	N/A
Critical Commercial Assays		
Direct-zol RNA Miniprep kit	Zymo Research	Cat#R2052
NEBNext Ultra II Directional RNA Library kit	New England Biolabs	Cat#E7765S
Qubit dsDNA BR Assay Kit	ThermoFischer	Cat#Q32850
Animal Tissue DNA Isolation Kit	Bionano Genomics	Cat#80002
DLS DNA Labeling Kit	Bionano Genomics	Cat#80005
Saphyr Chip G1.2	Bionano Genomics	Cat#20319
In-Fusion HD Cloning Plus	Clontech	Cat#638911
TransIT-LT1 Transfection Reagent	Mirus Bio	Cat#MIR2306
Deposited Data		
Raw and analyzed data (both)	This paper	GEO: GSE157428
Experimental Models: Cell Lines		
Mouse Neuro2A Cells	Olmsted et al., 1970	ATCC #CCL-131; RRID:CVCL_0470
Oligonucleotides		
See Table S7.	This paper	N/A
Recombinant DNA		
Plasmid: pEGFP-C1	Clontech	Cat#6084-1
Plasmid: pcDNA3.1	Invitrogen	CAT#V790-20
Plasmid: pcDNA3.1-BicD2-KIF1Balpha	This paper	N/A
Plasmid: pcDNA3.1-V5-His-TOPO	Invitrogen	Cat#K4800-01
Plasmid: FLAG-BicD2-KIF5A	This paper	N/A
Plasmid: FP-GRIP1-KBD +exon 21	This paper	N/A
Plasmid: FP-GRIP1-KBD −exon 21	This paper	N/A
Plasmid: BicD2-FKBP	Gift from Gary Banker, Bentley and Banker, 2015	N/A
Software and Algorithms		
FASTQC	Andrews, 2010	https://www.bioinformatics.babraham.ac.uk/projects/fastqc/
STAR	Dobin et al., 2013	https://github.com/alexdobin/STAR
Kallisto	Bray et al., 2016	https://pachterlab.github.io/kallisto/about
Sleuth	Pimentel et al., 2017	https://pachterlab.github.io/sleuth/about
MISO	Katz et al., 2010	https://miso.readthedocs.io/en/fastmiso/
Pymc3	Salvatier et al., 2016	https://docs.pymc.io/
Revigo	Supek et al., 2011	http://revigo.irb.hr/
SignalP	Nielsen et al., 1997	http://www.cbs.dtu.dk/services/SignalP/
ImageJ/Fiji	Schindelin et al., 2012	https://imagej.net/Fiji

## Highlights

- DM1 RNA-seq shows mis-splicing of neurotransmitter receptors along a severity gradient
- Repeats >1,000 CTGs occur in all individuals; lengths correlate with mis-splicing
- GRIP1 mis-splicing perturbs kinesin association and may alter synaptic trafficking
- Expression changes suggest neuroinflammation and downregulation of neuronal genes
