# Supplementary material for: Transcriptome alterations in myotonic dystrophy frontal cortex
Source: Cell Rep. Author manuscript; Available in PMC 2022 Jul 11. (PMC9272850; doi:10.1016/j.celrep.2020.108634)
Supplement: Document S1 [file NIHMS1821080-supplement-Document_S1.pdf]

Cell Reports, Volume *34*

Supplemental Information

Transcriptome alterations in myotonic dystrophy frontal cortex

Brittney A. Otero, Kiril Poukalov, Ryan P. Hildebrandt, Charles A. Thornton, Kenji Jinnai, Harutoshi Fujimura, Takashi Kimura, Katharine A. Hagerman, Jacinda B. Sampson, John W. Day, and Eric T. Wang

Figure S1

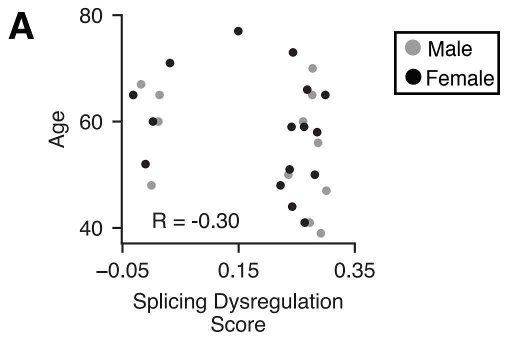

Figure S1 (Related to Fig. 1-2). Splicing dysregulation does not correlate with age or show sex bias. A) Scatter of patient ages versus splicing dysregulation score with Pearson R = -0.30. Sex of each individual is indicated by differences in color; males are in gray and females in black. B) Histograms of CTG repeat lengths as assessed by Bionano Saphyr. C) Histogram of measurements merged from all 7 DM1 samples studied.

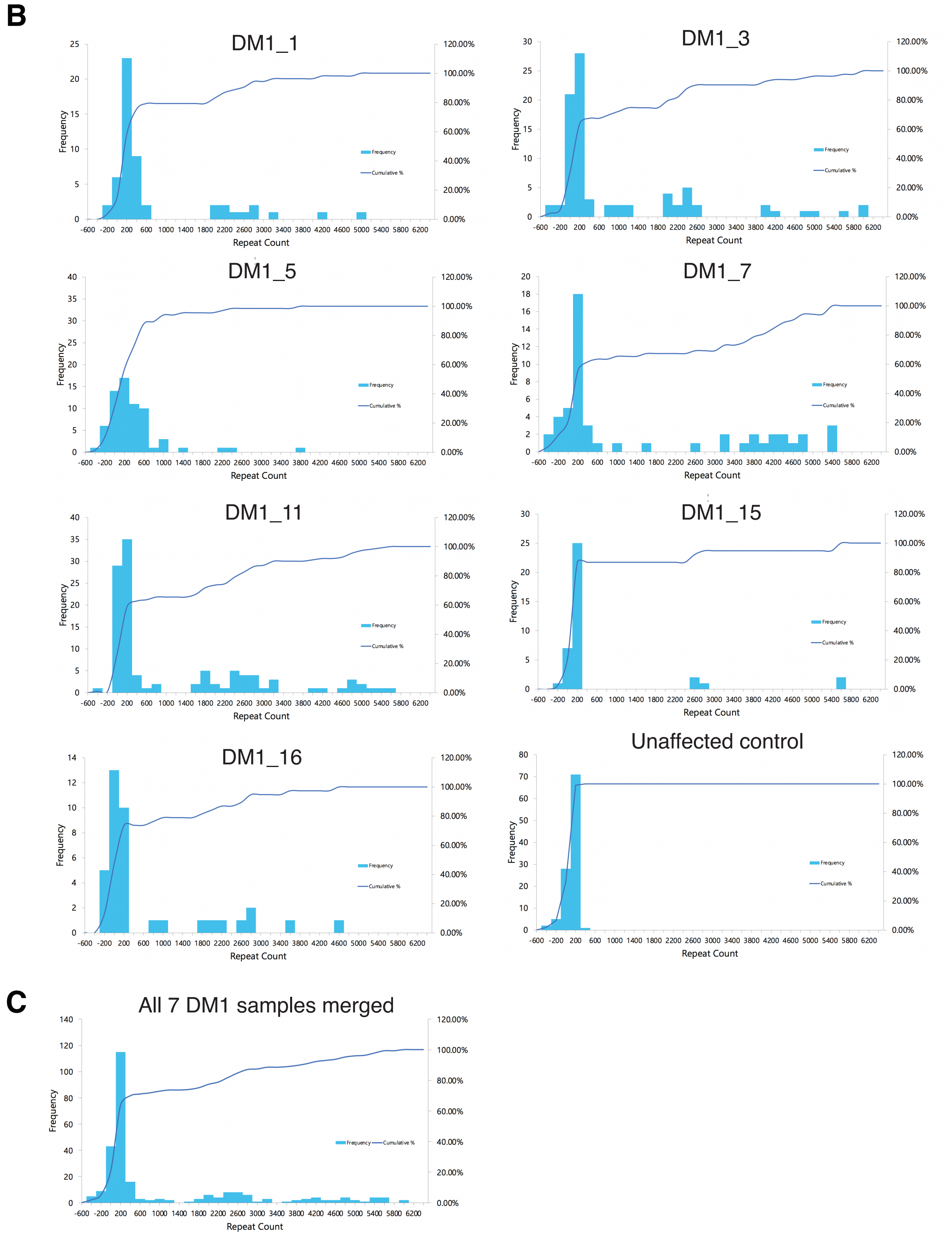

Figure S2

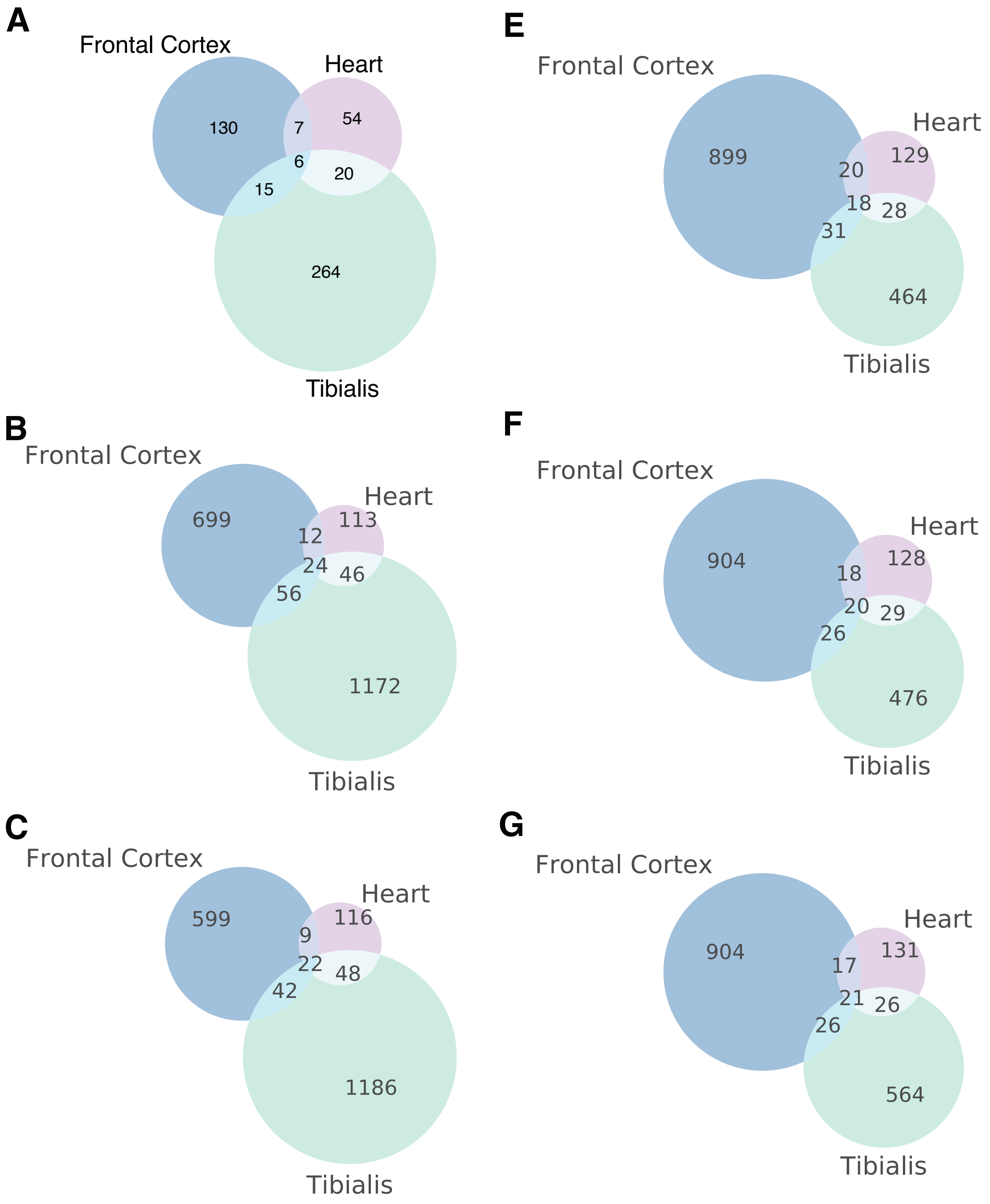

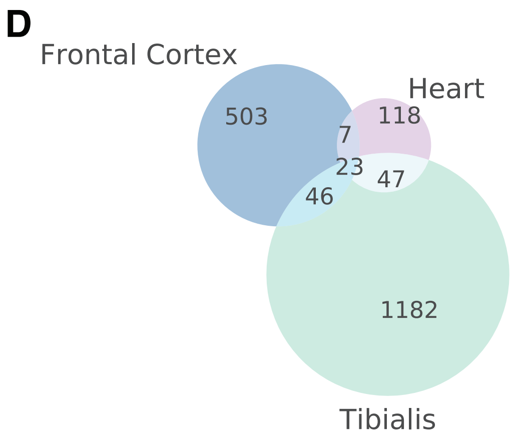

Figure S2 (Related to Fig. 4). Patient cohort size largely affects the number of mis-spliced transcripts captured. A-C) Three separate random subsamples of the DM1 FC cohort were used to compute the number of exons dysregulated in FC, TA, and heart. 12 DM1 FC samples and 6 unaffected FC samples were selected in each subsample. All subsamples show that 15-19% of events significant in heart are also significant in FC, using $|\Delta\psi| \geq 0.1$ and $p \leq 0.01$ (rank-sum test). Across the subsampling runs, the number of events identified to be significantly dysregulated in DM1 FC was 59-82% of the total 968 identified when using all samples, as shown in Figure 4A. D-F) Three separate random subsamples of the DM1 TA cohort were used to compute the number of exons dysregulated in FC, TA, and heart. All comparisons show that 23-26% of events significant in heart are also significant in TA, using $|\Delta\psi| \geq 0.1$, $p \leq 0.01$ (rank-sum test). Across the subsampling runs, the number of events identified to be significantly dysregulated in DM1 TA was 41-50% of the total 1298 identified when using all samples, as shown in Figure 4A.

Figure S3

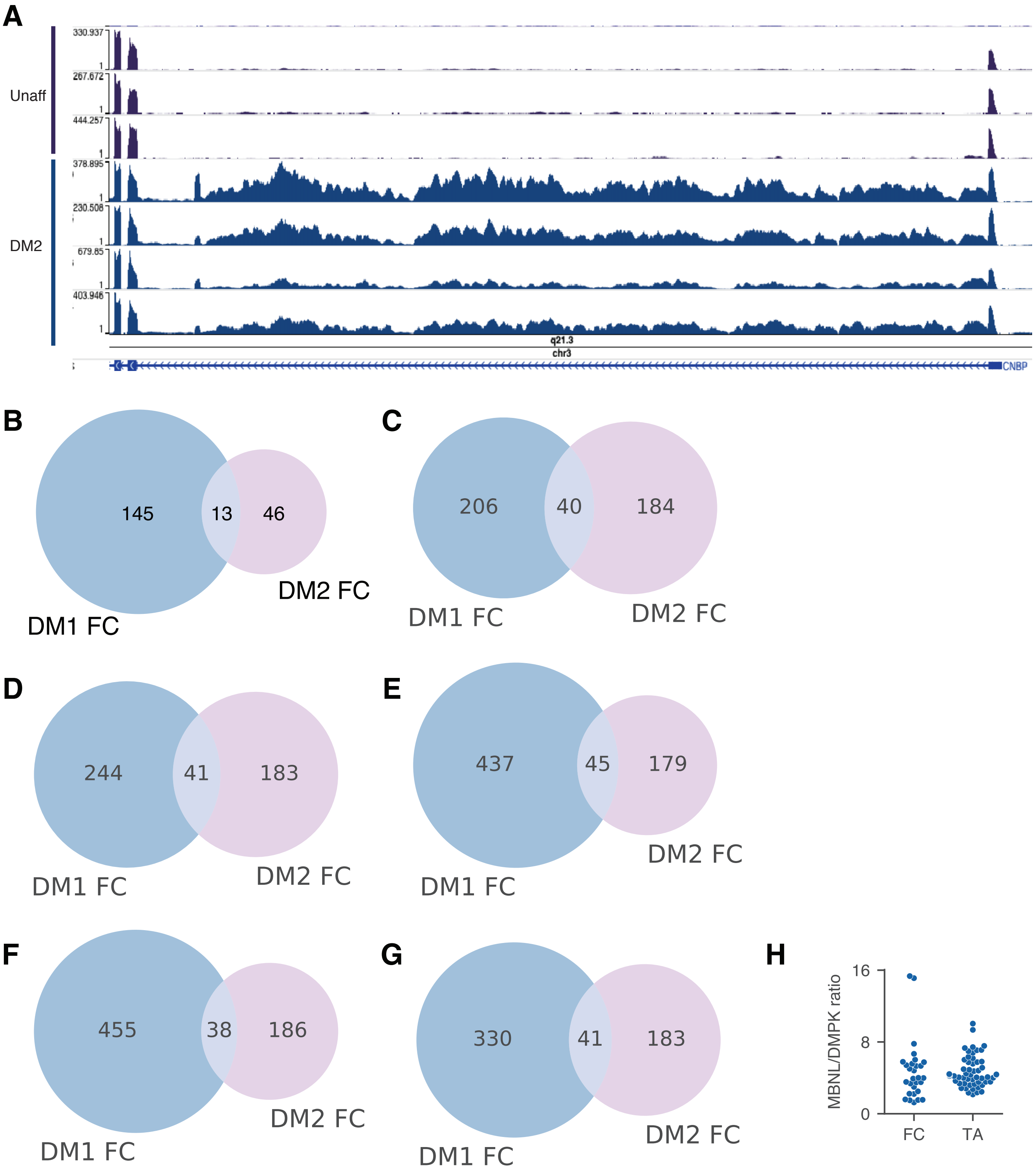

Figure S3 (Related to Fig. 5). Small patient cohort size of DM2 limits global mis-splicing captured. A) Intron 1 retention in CNBP is observed in DM2 samples, but not in 3 representative unaffected controls. B) Overlaps computed using $|\Delta\psi| \geq 0.2$, $p \leq 0.01$ (rank-sum test). C-E) Three separate random subsamples of the DM1 FC samples (4 DM1 patients, 8 unaffected controls) were used to compute the number of exons dysregulated in DM1, DM2, or both. All sub-samples show that 17-20% of the events dysregulated in DM1 were also dysregulated in DM2, using $|\Delta\psi| \geq 0.1$, $p \leq 0.01$ (rank-sum test). The number of events significantly regulated in DM1 FC samples ranged between ~25-50% of the total 968 identified when using all samples, as shown in Figure 5A. F) Overlap of events when analyzing the 4 least severe DM1 patients (according to total splicing dysregulation) versus DM2 patients. G) Overlap of events when analyzing the 4 most severe DM1 patients (according to total splicing dysregulation) versus DM2 patients. H) Ratio of DMPK to MBNL TPMs in frontal cortex and tibialis anterior.

Figure S4

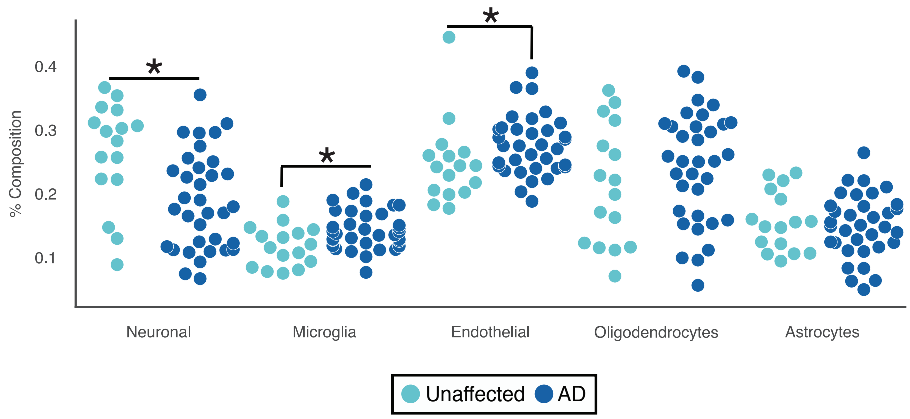

Figure S4 (Related to Fig. 6). Cell composition analysis of the entorhinal cortex in AD patients shows significant neuronal loss, with increases in microglial and endothelial composition. RNA-seq data derived from 34 Alzheimer's patients and 16 unaffected control entorhinal cortex samples were analyzed. We estimated cell type composition using our Bayesian Inference approach. By rank-sum test, AD patients were found to have significantly lower neuronal composition and significantly higher microglial and endothelial cell composition.
